# Increasing Trends of Leptospirosis in Northern India: A Clinico-Epidemiological Study

**DOI:** 10.1371/journal.pntd.0000579

**Published:** 2010-01-12

**Authors:** Sunil Sethi, Navneet Sharma, Nandita Kakkar, Juhi Taneja, Shiv Sekhar Chatterjee, Surinder Singh Banga, Meera Sharma

**Affiliations:** 1 Department of Medical Microbiology, Post Graduate Institute of Medical Education and Research (PGIMER), Chandigarh, India; 2 Department of Internal Medicine, PGIMER, Chandigarh, India; 3 Department of Histopathology, PGIMER, Chandigarh, India; Instituto Butantan, Brazil

## Abstract

**Background:**

Leptospirosis, a zoonosis associated with potentially fatal consequences, has long been a grossly underreported disease in India. There is no accurate estimate of the problem of leptospirosis in non-endemic areas such as north India.

**Methods/Principal Findings:**

In order to understand the clinical spectrum and risk factors associated with leptospirosis, we carried out a retrospective study in patients with acute febrile illness in north India over the last 5 years (January 2004 to December 2008). There was increased incidence of leptospirosis (11.7% in 2004 to 20.5% in 2008) as diagnosed by IgM ELISA and microscopic agglutination titer in paired acute and convalescent sera. The disease showed a peak during the rainy season (August and September). We followed up 86 cases of leptospirosis regarding their epidemiological pattern, clinical features, laboratory parameters, complications, therapy, and outcome. Mean age of patients was 32.6 years (2.5 years to 78 years) and males (57%) outnumbered females (43%). Infestation of dwellings with rats (53.7%), working in farm lands (44.2%), and contact with animals (62.1%) were commonly observed epidemiological risk factors. Outdoor workers including farmers (32.6%), labourers (11.6%), para-military personnel (2.3%), and sweepers (1.2%) were commonly affected. Modified Faine's criteria could diagnose 76 cases (88.3%). Renal failure (60.5%), respiratory failure (20.9%), the neuroleptospirosis (11.6%), and disseminated intravascular coagulation (DIC) (11.6%) were the commonest complications. Five patients died, giving a case fatality rate of 5.9%.

**Conclusions/Significance:**

There has been a rapid rise in the incidence of leptospirosis in north India. Severe complications such as renal failure, respiratory failure, neuroleptospirosis, and DIC are being seen with increasing frequency. Increased awareness among physicians, and early diagnosis and treatment, may reduce mortality due to leptospirosis.

## Author Summary

Leptospirosis is often not suspected by physicians in patients with acute febrile illnesses reporting from supposedly “non-endemic areas,” including north India. Clinical manifestations are protean, and complications can affect most organ systems, including liver, kidneys, lungs, and the central nervous system. Timely diagnosis and specific therapy can reduce severity of illness and, in turn, mortality. In this study conducted at a tertiary care center in north India, we find how a much-neglected disease entity has emerged as a major cause of acute febrile illness in a so called “non-endemic area.” Incidence is increasing yearly. The majority of patients were from a rural background, and were farmers or farm labourers. Poor hygiene, contact with animals, rat infestation of houses, and contact with stagnant dirty water are the major determinants of disease. Apart from the usual symptoms of intermittent fever with chill and rigor, hepatosplenomegaly, renal decompensation, muscle pain and tenderness, and conjunctival suffusion, signs and symptoms indicating involvement of the respiratory and central nervous systems were also commonly observed. Severe complications resulting in mortality do occur and is especially due to late suspicion among primary level physicians, and the resulting inappropriate therapy.

## Introduction

Leptospirosis, a worldwide zoonosis associated with sinister complications and fatalities, has been recognized in India since 1931 [1]. It is especially rampant in southern, central, eastern and western India, where heavy monsoon, animal rearing practices, unplanned urbanization and agrarian way of life predispose to this infection [1],[2],[3],[4],[5],[6],[7]. Leptospirosis has long been recognized as one of the foremost causes of acute febrile illness in those parts of the country [2],[5]. Though similar conditions exist in the north, reports of this disease from north India are few and only of recent origin (two of them from our center) [8],[9],[10],[11],[12]. Lack of awareness, clinical suspicion and active surveillance could be the probable reason. Leptospirosis has been a neglected disease even in developed countries like USA [13]. There is a wide spectrum of clinical presentations for leptospirosis. While most patients with *Leptospira* infection present only with mild fever and recover without complications, a small proportion develops various complications due to involvement of multiple organ systems. We have previously highlighted the importance of leptospirosis in pyrexia of unknown origin (PUO) cases [8],[9] in our center (Post Graduate Institute of Medical Education and Research, Chandigarh, India) situated in north India. Being a tertiary care center, undiagnosed and complicated patients are referred from all across the northern states.

Recently we observed an increase in the number of leptospirosis cases. We retrospectively reviewed the records of 86 such cases and observed the varied clinical manifestations and course of the disease in these patients. Apart from usual cases of acute icteric and anicteric febrile illnesses, severe manifestations of the disease, like neuroleptospirosis, haemorrhagic pneumonitis, and adult respiratory distress syndrome, were observed.

## Materials and Methods

This present study is a retrospective review of records of leptospirosis cases diagnosed at our institute during the last 5 years (2004 to 2008). During this period, the microbiology laboratory received 1391 blood samples from suspected cases with pyrexia of unknown origin for leptospira serology. Paired acute and early convalescent (10–15 days into illness) serum samples were tested for specific anti-leptospira IgM antibody using the PanBio IgM ELISA (Panbio diagnostics, Brisbane, Australia). The test procedure was performed according to the protocol provided along with the kit. The results were interpreted according to manufacturer's instructions, i.e. values <9 PanBio ELISA units were considered negative, 9–11equivocal, and >11 positive. For samples showing equivocal results, another blood sample was drawn after a period of 10 days, and the test was repeated. Negative and positive controls were kept with each test run. Microscopic agglutination test (MAT) could be done in a few samples only (paired sera). For this test these samples were sent to the National Reference Laboratory for Leptospirosis, Port Blair, Andaman Islands, India. MAT was carried out following standard procedure [14] using 10 live leptospiral reference strains as antigens. The strains belonged to serogroups *Australis*, *Autumnalis*, *Ballum*, *Bataviae*, *Canicola*, *Grippotyphosa*, *Icterohaemorrhagiae*, *Javanica*, *Pomona*, and *Tarassovi*. The criteria for a positive MAT test was a titre of ≥1∶400 in a single sample, four-fold rise in titre or seroconversion in paired samples. Partial autopsy was done on one of the five patients who died, after obtaining informed written consent from close relatives. Representative tissue samples were processed for paraffin embedding and histological evaluation.

Eighty six patients positive for leptospira serology were evaluated noting their clinical history, presentation, radiological features, laboratory parameters, management, course and outcome. Utilizing clinical, epidemiological, and laboratory parameters modified Faine's criterion was scored and assessed [13]. Scoring system by modified Faine's criteria is detailed in Table 1.

**Table 1:** Modified Faine's criteria.

**Part A: Clinical Data**	**Score**
Headache	2
Fever	2
If fever, temperature 39°C or more	2
Conjunctival suffusion (bilateral)	4
Meningism	4
Muscle pain (especially calf muscle)	4
Conjunctival suffusion+Meningism+Muscle pain	10
Jaundice	1
Albuminuria or nitrogen retention	2
**Part B: Epidemiological Factors**	**Score**
Rainfall	5
Contact with contaminated environment	4
Animal contact	1
**Part C: Bacteriological and Laboratory Findings**	
Isolation of *Leptospira* on culture	Diagnosis certain
Positive serology	
ELISA IgM positive*; SAT positive*; MAT single high titre* (Any one of the three tests should be scored)	15
MAT rising titre (paired sera)	25

A presumptive diagnosis of leptospirosis may be made if: (i) Score of Part A+Part B = 26 or more (Part C laboratory report is usually not available before fifth day of illness; thus it is mainly a clinical and epidemiologic diagnosis during early part of disease) or Part A+Part B+Part C≥25.

A score between 20 and 25: Suggests a possible but unconfirmed diagnosis of leptospirosis.

The study was approved by the Institute ethical committee, PGIMER, Chandigarh. Informed consent for blood samples and for autopsy was not needed in the study. Blood samples are received in the laboratory routinely as part of patient care. Autopsies are routinely done at our center; institute ethical committee approval is not needed for the autopsy because it was not done for research purposes.

## Results

**Figure 1:**
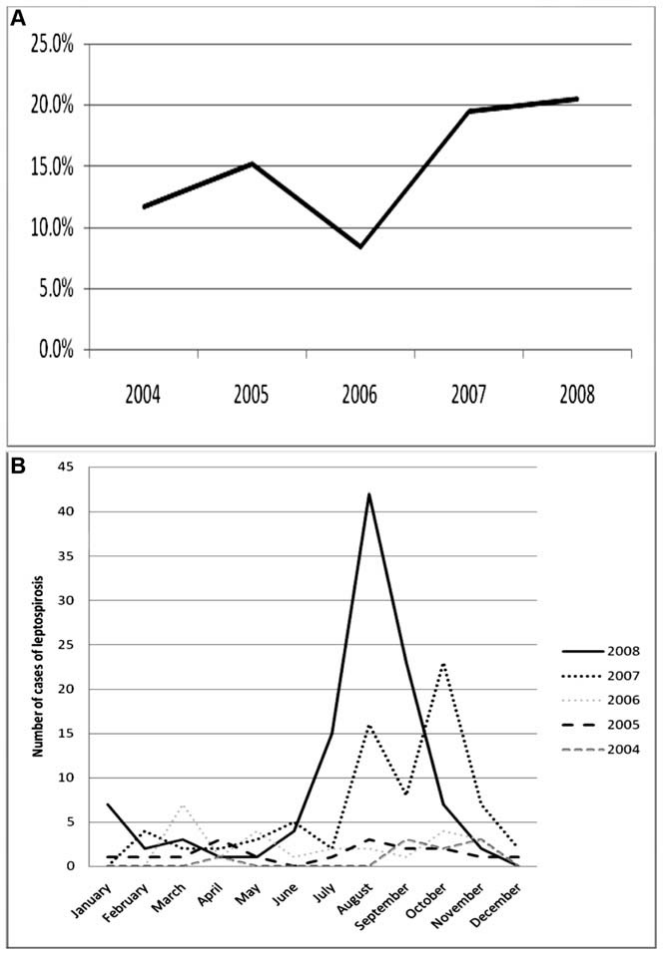
Time trends in leptospirosis. (A) Percentage of leptospirosis patients among those with acute febrile illnesses. (B) Month- and year-wise distribution of cases. * Total number of leptospirosis patients was 232 (9 in 2004, 17 in 2005, 25 in 2006, 74 in 2007, and 107 in 2008).

**Figure 2:**
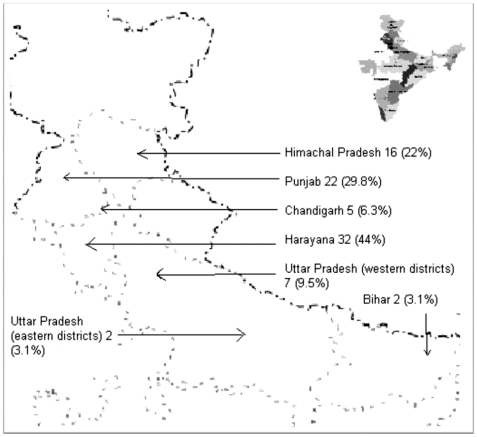
Area of residence of 86 patients of leptospirosis in India.

During the study period, from 2004 to 2008, there was a sustained rise of leptospirosis cases from (11.7% to 20.5%) (Figure 1A). In all we detected 232 cases of leptospirosis in the five years of study period (9 in 2004, 17 in 2005, 25 in 2006, 74 in 2007, and 107 in 2008). Cases were more common in the months of July-October for most of the years (Figure 1B). The patients resided in different states of north India, however majority of our patients were from Punjab, Haryana and Himachal Pradesh (Figure 2). Mean age of patients was 32.6 (±0.7) years with a range from 2.5 years to 78 years. Male patients (49, 57%) outnumbered female patients (39, 43%). Most of the patients (∼70%) were young adults in their 2^nd^, 3^rd^, and 4^th^ decades of life (Table 2). Sixty six (76.7%) patients were from rural areas, travelling to endemic area was suggestive in 2 patients (Table 2). Major epidemiological risk factors noted in our patients include wet environmental living conditions, lack of protective footwear, infestation of dwelling with rats, working in farm lands, contact with animals, especially cattle, bathing in public places, history of unprotected contact with dirty stagnant water, alcohol addiction, and smoking (Table 3). Most of the patients by occupation were farmers (28, 32.6%), followed by housewives (19, 22.1%), students (11, 12.8%), labourers (10, 11.6%), indoor non-manual workers (10, 11.6%), para-military personal (2, 2.3%), sweeper (1, 1.2%), carpenter (1, 1.2%); and 4 were children below 4 years (unemployed); two of the female patients were pregnant.

**Table 2:** Epidemiological pattern and age group of 86 leptospirosis patients.

Epidemiological pattern	Subcategory	Number (%)
Rural leptospirosis		66 (76.7)
Urban leptospirosis		20 (23.3)
Probable recreational leptospirosis cases*		2 (2.3)
Age	≤10 years	4 (4.7)
	11–20 years	17 (19.8)
	21–30 years	25 (29.1)
	31–40 years	18 (20.9)
	41–50 years	12 (14)
	51–60 years	6 (7)
	61–70 years	3 (3.5)
	>70 years	1 (1.2)

*: Documented history of travel to known endemic areas alongwith history of unprotected bathing in ponds of those areas.

**Table 3:** Epidemiological risk factors found in the 86 leptospirosis patients.

Risk factor	Subcategory	Number (%)
Wet surroundings, streams nearby, dirty surroundings		53 (61.8)
Use of public bathing facilities, ponds		32 (36.7)
Dwelling	Mud/thatch houses	14 (16.3)
	Kucha pucca (part mud and part brick)	16 (18.4)
	Pucca (brick) houses	56 (65.3)
	Rat infestation	46 (53.7)
Animal contact	Any animal (Total)	53 (62.1)
	Cattle	48 (56.1)
	Sheep	1 (1.5)
	Poultry	1 (1.5)
	Dogs	4 (4.5)
	Goat	4 (4.5)
	Rodent	1 (1.5)
Not wearing shoewear		31 (36.5)
Works in fields		38 (44.2)
Alcoholic		28 (32.7)
Smoker		14 (16.3)
Entered water logged area barefoot		11 (12.8)

Fever was seen in all 86 individuals, being intermittent and associated with chills and rigor in most of the patients. Icterus, abdominal pain, hepatomegaly, muscle pain and tenderness, headache, vomiting, breathlessness, splenomegaly, subconjunctival effusion, oliguria and altered sensorium were the common manifestations of the disease. Meningism, lymphadenopathy, arthralgia were seen in fewer cases (Table 4). The most frequently observed alterations in laboratory parameters in these patients included leukocytosis, anemia, thrombocytopenia, elevated hepatic enzymes [alanine aminotransferase (ALT) and asparate aminotransferase (AST)] in the range of 100 to 200 IU/dl, elevated serum bilirubin levels in the range of 2–8 mg/dl, thrombocytopenia, increased prothrombin time, and D-dimer positivity (Table 5). Laboratory parameters suggestive of DIC (derangement of any three of prothrombin time, activated partial thromboplastin time, D-dimer, thrombin time, fibrinogen level, fibrinogen degradation products, and fragmented RBCs on a peripheral blood film+a low platelet count) were present in ten patients. Modified Faine's criteria could diagnose 76 cases (88.3%).

**Table 4:** Clinical features of 86 leptospirosis patients.

Clinical feature	Subcategory	Number (%)
Fever (≥38°C)	In any form	86 (100)
	With chills and rigor	52 (60.5)
	Intermittent	82 (95.3)
	Continuous	4 (4.7)
Headache		32 (37.2)
Myalgia with muscle tenderness		26 (30.2)
Jaundice		63 (73.3)
Abdominal Pain		30 (34.9)
Hepatomegaly		46 (53.5)
Vomitting		34 (39.5)
Respiratory symptoms	Cough (dry or with mucoid expectoration)	13 (15.1)
	Breathlessness	27 (31.4)
Splenomegaly		23 (26.7)
Oliguria		25 (29.1)
Conjunctival suffusion		16 (18.6)
Neurological manifestations	Altered sensorium	33 (38.4)
	Meningism	6 (7)
	Generalized tonic-clonic seizures	3 (3.5)
	Headache	32 (37.2)
	Focal neurological deficit	2 (2.3)
Lymphadenopathy		5 (5.8)
Arthralgia		6 (7)
Diarrhoea		10 (11.6)
Bleeding manifestations	Epistaxis	1 (1.2)
	Hematuria	4 (4.7)
	Hemoptysis	1 (1.2)
	Petechiae	9 (10.5)
	Hematemesis	4 (4.7)
Pitting edema		10 (11.6)
Maculo-paupar rash		3 (3.5)
Acneform rash		1 (1.2)

**Table 5:** Laboratory parameters of 86 patients at time of diagnosis and hospital stay.

Laboratory parameter	Number (%)
Leukocytosis >11000/mm^3^	53 (61.6)
Anemia (Hb<10.0 gm/dl)	49 (57)
D-dimer positive	6 (7)
Increased Prothrombin Time	8 (9.3)
Thrombocytopenia (<100000/dl)	16 (18.6)
Deranged asparate transaminase, alanine transaminase *	70 (81.4)
Hyperbilirubinemia**	66 (76.7)

*: In 68 cases ranged from 60 IU to 200 IU, in two cases was >200 IU.

**: In 64 cases, between 2–8 mg/dl, in 2 cases >8 mg/dl.

By far the commonest complication was renal failure (serum creatinine >1.4 mg/dl). Other common complications observed among these patients included respiratory failure requiring mechanical ventilation, neuroleptospirosis, ascitis and pleural effusion. Laboratory confirmation of disseminated intravascular coagulation (DIC) could be noted in 10 cases; however, obvious external bleeding, petechiae and echymoses were seen in 19 cases (Table 6). Majority of complications occurred in the second week of illness or later, however 18 cases of renal impairment (34.5% of 52), three cases of respiratory failure (16.7% of 18) and one case of neuroleptospirosis (10% of 10) occurred in the first week of illness. Three cases of mixed infection with *P. vivax*, 2 with *P. falciparum*, one each with dengue and hepatitis A virus were also observed. No specific clinical feature or complication correlated with the geographic area of incidence of the disease.

**Table 6:** Complications of leptospirosis cases while in hospital.

Complications	Number (%)
Renal failure (serum creatinine >1.4 mg/dl)	52 (60.5)
Respiratory failure requiring mechanical ventilation	18 (20.9)
Hemorrhagic pneumonia (autopsy proven)	1 (1.2)
Neuroleptospirosis (with CT evidence of diffuse cerebral edema or neurological deficit)**	10 (11.6)
Pleural effusion*	10 (11.6)
Ascitis*	14 (16.3)
Disseminated intravascular coagulation (DIC)	10 (11.6)

*: Mild ascitis and mild to moderate pleural effusion mainly detected in chest X-ray and ultrasonographic investigations.

**: 33 cases presented with altered sensorium and 32 with headache. 10 cases of these cases could be definitely categorized as neuroleptospirosis, as evidenced by CT finding of diffuse cerebral edema, generalized seizures, neck rigidity, or neurological deficits.

**Figure 3:**
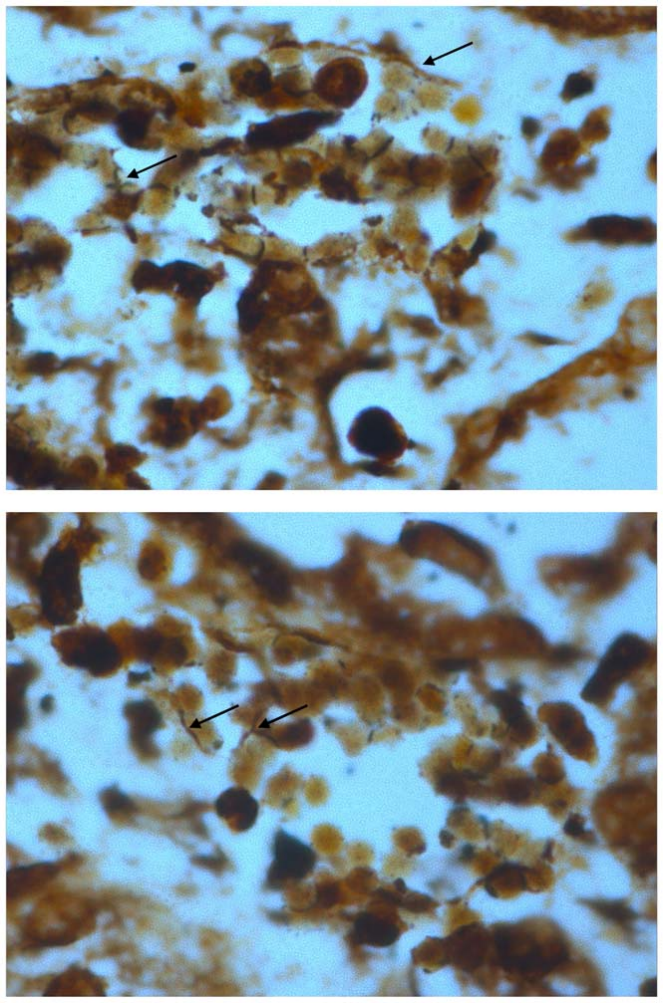
Warthin Starry stain showing *Leptospira* in the tubular cells of the kidney.

Treatment with once a day ceftriaxone therapy was given to 66 cases, doxycycline therapy alone to 3 patients; and combined doxycycline and ceftriaxone therapy to 17 patients. All these patients had documented hospital acquired septicemia or were suspected of having such superinfection. High dose corticosteroid therapy was instituted in 7 cases, all of them with respiratory failure. Six of them survived. Of all 86 cases, 5 patients died (5.9%). Pathological autopsy was done in one of these cases. Leptospires were demonstrated in post-mortem kidney (Figure 3) and lung specimens of the patient using the Warthin-Starry stain. By the microscopic agglutination test the following serovars gave highest titres (>1∶400) against patients' sera: *Pomona*, *Ballum*, *Gryppotyphosa*, and *Autumnalis*.

## Discussion

The increase in leptospirosis cases during the last few years is possibly the result of greater awareness of this disease in the north and more drier parts of the country. WHO estimates the incidence of leptospirosis between 0.1–1 cases/100000 population/year in temperate, non-endemic areas and between 10–100 cases/100000 population/year in humid, tropical, endemic areas. Though north India receives less rainfall compared to the coastal regions and the south, most areas still receive ≥100 cm rainfall in the monsoon season between July and October. Flooding and unseasonal heavy precipitation are not uncommon like the August floods in Punjab, Bihar and Himachal Pradesh in 2008. A large proportion of the population depends on the agrarian way of life. Intimate contact with animals, unprotected entry into waterlogged fields, and bathing in contaminated community ponds are a part of rural life in across north India [2]. These are precisely the conditions most suitable for the survival and transmission of *Leptospira* [2]. Thus the stupendous rise in the number cases seen in this study should not come as a surprise. Also, previous reports from Chandigarh, Ludhiana, New Delhi, and Uttar Pradesh point to the fact that leptospirosis is present all over India [8],[9],[10],[11],[12]. Reports from Italy, Bulgaria and certain centers in south India point at a decreasing incidence of leptospirosis, this is however, not true at our center [15]. Like our study, a study conducted in Chennai (south India) too has seen a rapid increase in leptospirosis cases between 2004 and 2006 [3].

We used the Pan Bio IgM ELISA to screen for leptospirosis in well timed acute and convalescent blood samples, and performed MAT on few samples. It is unlikely that a large number of leptospirosis cases were missed since sensitivity of PanBio IgM ELISA as used in this study has been reprted to be as high as 76–90% [16],[17]. Though dark ground microscopy and culture in EMJH media may be performed from blood and urine samples during the acute phase of the disease, these have relatively poor sensitivity in detection of the disease [1]. In India, MAT is performed only in the reference laboratory at Andaman. Most laboratories hence prefer IgM ELISA formats for the diagnosis of leptospirosis [1]. Further this test is reactive even in early cases of leptospirosis when MAT may be negative [1].

The modified Faine's criteria could diagnose leptospirosis in 76 patients. MAT being unavailable in our center, the original Faine's criteria is not an option for our physicians. Instead the modified Faine's criterion is a useful guide. Sivakumar S et al., 2004 modified the original Faine's criteria to include local factor like rainfall, and newer investigations like IgM ELISA and Slide Agglutination test (SAT) [13]. No modifications were however made to the clinical criteria. Rainfall has been added because of the observation that most cases of leptospirosis are reported in monsoon and post-monsoon period. Compared to MAT, IgM ELISA and SAT are simpler and more sensitive tests that can be used to diagnose acute leptospira infections including milder forms which are associated with low clinical scores [13],[18]. The differential diagnosis of leptospirosis is very long, and this disease easily confuses with other viral, parasitic and bacterial infections. We suggest that the modified criterion be used by physicians in this regard.

Farmers and farming labourers (32.6% and 11.6% in our study) are the ones most commonly infected in the rural setting and the disease is associated with sowing and harvesting seasons and meteorological phenomena like monsoons. Presence of farming animals, and rodents, some of them leptospiral carriers, in the farmland, wet and humid environmental conditions for *Leptospira* survival, and frequent human agricultural and animal rearing activity form the core determinants of *Leptospira* transmission [2]. That leptospirosis occurs in those living in unhygienic conditions was evident when 34.7% of the patients lived in mud and part-mud houses and 12.8% gave history of entering waterlogged areas barefoot.

Intermittent fever with chills and rigor was the most common manifestation; however continuous fever and lack of chills and rigor were also seen in a few patients. Though the incidence of icteric and severe disease with renal failure has decreased in certain centers of south India [15], the present study found several cases manifesting with severe icteric disease and renal failure. Prabhakar MR *et al.* point out that epidemiological and clinical pattern of infectious disease change in course of time and leptospirosis is no exception to this rule [15]. We further argue that the pattern may vary from region to region. Knowledge of such changing epidemiological and clinical profile of leptospirosis is essential for successful prevention, early diagnosis and treatment [15].

Typically a biphasic illness, complications ensue in the second immune phase of the disease [1],[19]. Renal failure is the commonest complication noted, both in anicteric and icteric leptospirosis [1]. Azotemia, oliguria and anuria commonly occur during the second week of illness, but may appear as early as 3 to 4 days after onset [1].

Cough, pleural effusion, respiratory failure, and hemorrhagic pneumonia were commonly observed in our study. Respiratory symptoms are known to occur commonly in severe leptospirosis [19],[20],[21]. However, when respiratory complications are predominant, chances of misdiagnosis as community acquired pneumonia increase. Pulmonary complications have especially been noted to occur early and more frequently in the Andamans, and has been associated with higher mortality [21]. The higher frequency of respiratory presentation is in contrast to our earlier studies [8],[9]. Many patients with severe respiratory problems progress to develop multiple organ dysfunction syndrome and are admitted in intensive care [20] resulting in further complications from hospital acquired infections.

Majority of our patients with a diagnosis of neuroleptospirosis presented with an encephalitic syndrome, with altered sensorium, and headache. A minority also had signs of meningism, and experienced generalized seizures. This finding is similar to the case series reported by Mathew P et al., 2006 [22]. Neurological manifestations were seen in 10%–15% of leptospirosis patients [22]. Such manifestations are varied and often lead to misdiagnosis, unless strongly suspected. Most frequent manifestations include altered sensorium and neck stiffness [22]. More importantly leptospirosis is responsible for 5%–13% of all cases of aseptic meningitis [23]. Generalised tonic-clonic seizures with altered sensorium, encountered in 3 patients in our study, are a manifestation of the encephalitis [23]. Less common manifestations include hemiplegia [22],[23], intracranial bleed [23], cerebellitis [23], movement disorder [23], myelitis [23], acute flaccid paralysis including Guilain Bare syndrome [23], mononeuritis [23], facial palsy [22],[23], and neuralgias [23]. CT scan may be normal [22], however diffuse cerebral edema may be seen in a minority of patients. An early and specific diagnosis is mandatory as effective and specific therapy is available. Mathew P *et al.*, 2006 observe that neuroleptospirosis should be considered in the differential diagnosis of neuroinfections associated with hepatorenal dysfunction in endemic areas [22].

Thrombocytopenia is especially common, while minority of patients also present with prolonged prothrombin time due to hypoprothrombinemia [1],[23]. Activation of the coagulation system is an important feature of leptospirosis [23]. Direct or indirect signs of the DIC and intravascular platelet aggregation are characteristic of malignant forms of leptospirosis [23]. Concentrations of fibrinogen, D-dimer, thrombin-antithrombin III complexes, and prothrombin fragment 1,2 are significantly elevated in leptospirosis patients [23]. In our study, patients with leptospirosis had significantly longer prothrombin times (9.3%), were D-Dimer positive (7%), and had lower platelet counts (18.3%). In all 10 patients could be categorized as having DIC. Mild leukocytosis with a shift to the left is also commonly observed in leptospirosis [1],[23]. 61.6% of our cases had an increased leucocyte count.

These severe and unusual manifestations are often not recognized as leptospirosis, and other infectious etiology like viruses looked for. Such is especially common in areas wrongly thought to be non-endemic. This leads to delay in appropriate therapy and further progression of the disease. Further, renal complications can ensue even in the first week of illness [24], although they occur more often in the second or third week of illness as observed in our study. Including leptospirosis in the differential diagnosis and instituting early empirical therapy could reduce inadvertent deaths. Treatment with high dose corticosteroids in cases with severe complications, where the immune phase of disease has begun, is controversial [25]. Only 7 of our cases were put on high dose systemic corticosteroids. Further study on the role of systemic steroids in this disease is warranted. We also describe cases with unusually high ALT and AST levels (>200 IU/ml, 2 cases) and very high bilirubin levels (>8 mg/dl, 2 cases). Chronic alcoholism (32.7% in our study) may predispose to additional liver damage and symptomatic disease [2]. Coinfection with malaria, dengue and other viruses may present diagnostic dilemmas to the treating physician. Inevitably such cases have severe manifestations and result in high morbidity and mortality.

Once a day ceftriaxone therapy has been documented to have equal efficacy to penicillin therapy, and seemed to be the preferred therapeutic regimen in our center. The mortality in treated cases was high (5.9%), however it was comparable to that seen by Jayakumar M *et al.* (9.5%) [26]. Severe disease and mortality from *Leptospira* infection is not always due to the bacteria itself, but usually due to the destructive activity of the immune system of the host [1],[23],[25]. Before death, irreversible multiple organ dysfunction syndrome usually sets in, although leptospiraemia has ceased.

## Conclusion

A significant rise in the incidence of leptospirosis in north India was documented. Clinical manifestations and laboratory abnormalities were protean; severe complicated disease with renal or respiratory failure, neuroleptospirosis, and DIC was also observed. The increased awareness among physicians of protean clinical manifestations of leptospirosis and early laboratory diagnosis will help reduce morbidity and mortality associated with disease.
